# Preparation, Characterization, and Performance Evaluation of Petroleum Asphalt Modified with Bio-Asphalt Containing Furfural Residue and Waste Cooking Oil

**DOI:** 10.3390/polym14091683

**Published:** 2022-04-21

**Authors:** Shuo-Rong Lai, Shu-Jun Li, Yong-Li Xu, Wen-Yuan Xu, Xian-Quan Zhang

**Affiliations:** 1 Engineering Research Center of Advanced Wooden Materials, Ministry of Education, Northeast Forestry University, Harbin 150040, China; laishuorong@163.com (S.-R.L.); lishjun@nefu.edu.cn (S.-J.L.); 2 College of Civil Engineering, Northeast Forestry University, Harbin 150040, China; xuyongli77@163.com (Y.-L.X.); xuwenyuan@nefu.edu.cn (W.-Y.X.)

**Keywords:** furfural residue, waste cooking oil, bio-asphalt, partial substitution, road performance

## Abstract

The study aims to analyze the feasibility of proposing waste cooking oil and industrial waste furfural residue as raw materials to prepare bio-asphalt as partial substitutes for petroleum asphalt, so as to reduce the cost of pavement construction and decrease the consumption of non-renewable resources. In this study, 90# petroleum asphalt was partially substituted with the bio-asphalt in different proportions to prepare biomass-modified petroleum asphalt, the performance of which was first evaluated based on three indices: penetration, softening point, and ductility. Comparison of the crystal structures of the bio-asphalt and furfural residue were enabled by X-ray diffraction, and the blending mechanism and microscopic morphologies of the biomass-substituted asphalt mixtures were characterized by Fourier transform infrared spectroscopy and scanning electron microscopy. The results showed that the bio-asphalt was hydrophobic and exhibited excellent compatibility with 90# petroleum asphalt. The partial substitution of petroleum asphalt with bio-asphalt improved the low-temperature crack resistance of the asphalt by adversely affecting the high-temperature stability of the asphalt; however, when the bio-asphalt content was 8 wt.%, the performance parameters of the biomass-modified asphalt met the requirements of the 90# petroleum asphalt standard.

## 1. Introduction

Petroleum asphalt is a byproduct of crude oil processing and is widely used in highway road construction, as an anticorrosion coating, and for waterproofing materials. Considering the large consumption of fossil fuel resources and the harm of petroleum asphalt to both the environment and the health of construction workers, significant efforts are being made to replace or modify petroleum asphalt with renewable biomass [1,2,3].

Waste cooking oil refers to the oil that has failed to meet an edible standard after utilization. Approximately 5.6 million tons of waste cooking oils are being produced annually in China [4]. Most waste cooking oils are directly discharged into sewers and cause serious environmental pollution, some unscrupulous businesses even reuse them as edible oil after simple treatment, which will damage the health of consumers. Scientists have done a lot of research on the recovery and application of gutter oil. The most common application is to process waste cooking oil into products such as biodiesel, soap, animal feed, and feedstock for microbial growth [4,5,6,7]. The long-chain fatty acids contained in waste cooking oil are similar to the saturation fraction in asphalt, and they have good compatibility. Researchers found that the use of waste cooking oil in asphalt modification can reduce the use of asphalt mixing solvent and soften asphalt [8,9,10,11,12]. If waste cooking oil can be widely used in asphalt modification, it can not only improve the performance of asphalt pavement and reduce the cost of asphalt, but also provide a feasible scheme for the treatment of waste cooking oil.

Previous studies have analyzed the effects of modifying asphalt with cellulose and lignin, the two most abundant organic compounds in nature, on the mechanical properties of asphalt. The modification of asphalt with nanocellulose, which has a large specific surface area and high mechanical strength, improved the viscoelasticity and rutting resistance of asphalt [13,14,15]. Making use of the natural aromatic structure of lignin, lignin exhibited excellent compatibility with the polycyclic aromatic hydrocarbons in petroleum asphalt when mixed [16]. When petroleum asphalt was modified with lignin, the resulting bio-asphalt exhibited a higher thermal stability than the petroleum asphalt [17,18,19]; however, the lignin molecules could absorb the low molecular oil components in the asphalt, resulting in a sharp decrease in the thermal ductility of the modified asphalt [20,21].

**Figure 1:**
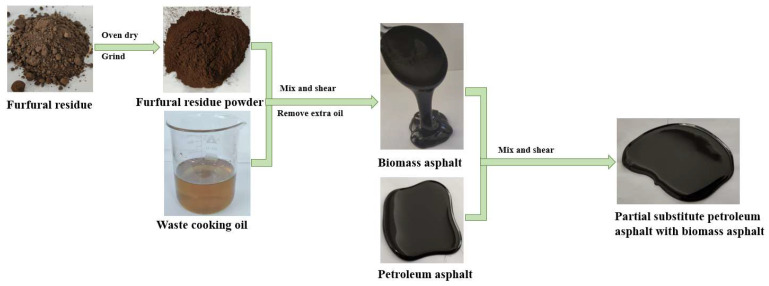
Scheme depicting the fabrication of the petroleum asphalt modified with bio-asphalt derived from furfural residue and waste cooking oil.

On an industrial scale, furfural is prepared by the acid-catalyzed dehydration of pentose sugars from hemicellulose in lignocellulosic raw materials (e.g., corncob and bagasse); however, the solid residue leftover after furfural production, known as furfural residue, still contains significant amounts of lignin, cellulose, and volatile organic compounds. In addition, hemicellulose is very hydrophilic and can be easily degraded by microorganisms; therefore, biomass without hemicellulose is more suitable for asphalt modification than biomass containing hemicellulose. In this study, after oven drying and grinding to a fine powder, the furfural residue was mixed with waste cooking oil, and the mixture underwent shearing to fabricate a bio-asphalt, after which petroleum asphalt was partially substituted with the bio-asphalt at concentrations of 4, 8, and 12 wt.%. A diagram of the preparation process is shown below in Figure 1. The basic performance of the biomass-modified asphalt mixtures was assessed by measuring the penetration, softening point, and ductility of the modified asphalts. In addition, the maximum substitution amount of the bio-asphalt needed to meet the standard requirements of 90# petroleum asphalt was determined. The effects of the bio-asphalt content on the high- and low-temperature performance of the modified asphalt mixtures was investigated by dynamic shear rheometry and bending beam rheometry. Finally, the high-temperature stability, low-temperature crack resistance, and water stability of the biomass-modified asphalts were determined by conducting rutting tests, low temperature bending tests, freeze-thaw splitting tests, and immersion Marshall tests to evaluate the utility of these bio-asphalt mixtures in road construction. The purpose of this paper is to evaluate the performance of petroleum asphalt modified by bio-asphalt prepared from furfural residue and waste cooking oil through these experiments, and to evaluate the feasibility of the prepared bio-asphalt as partial substitutes for asphalt.

## 2. Materials and Methods

### 2.1. Materials

The furfural residue used in the experiment was produced by Harbin Xingcheng Chemical Co., Ltd. (Harbin, China), in which the cellulose content was 47.63%, the hemicellulose content was 1.44%, the lignin content was 41.26%, and the ash content was 4.4% (all based on dry mass). To ensure that the furfural residue could be fully mixed with the waste cooking oil and asphalt, the residue was dried, ground, and passed through 80-mesh sieves.

Waste cooking oil (soybean oil) was acquired from local restaurants in Harbin. In this restaurant, the ingredients (chicken, french fries, fish, etc.), in batches of 100–200 g, were fried in approximately 4 L of soybean oil in a 6 L capacity fryer (BK-SD-81D, Sanding, Guangdong, China) for 5–8 min at a temperature of 175 ± 5 °C. The fried food in the mesh basket were then lifted for 1 min to allow adhering oil to drain back into the fryer. Additional batches were fried in the same manner every 10–15 min for a total of 6–8 h per day, adding about 500 mL of new oil the next day. After two days of being used, the oil is collected and then filtered with a 75 μm filter mesh to separate impurities.

The asphalt matrix used in this study was 90# petroleum asphalt.

### 2.2. Methods

#### 2.2.1. Analysis of the Raw Materials

Cellulose is the main component of furfural residue and is hydrophilic, making it potentially deleterious to the structural properties of asphalt, which is overall hydrophobic; hence, the water absorption rates of furfural residue were measured by storing at 20 °C and 90% relative humidity (RH) for 3 days. A STA 449F3 synchronous thermal analyzer was used to analyze the thermal stability of the furfural residue, which was heated at a rate of 10 °C/min from 30 °C to 700 °C under N_2_ at a flow rate of 20 mL/min. The pH and density of the waste cooking oil were measured as its basic properties. The TPM (total polar matter) content of waste cooking oil was determined following the AOCS Cd20-91 standard method. The pH value of waste cooking oil was measured by pH meter (PHS-3C, Shengci, Shang Hai, China). The density of waste cooking oil was measured by the liquid density meter (ET-03l, Yitenuo, Beijing, China). The technical indices of the 90# petroleum asphalt, such as penetration degree, softening point, and ductility, were measured according to the standards ASTM D5, D36, and D113, respectively.

#### 2.2.2. Preparation of the Bio-Asphalt

To ensure that the furfural residue did not deleteriously affect the low-temperature ductility of the asphalt by absorbing low molecular oil, the furfural residue needed to be fully swollen and uniformly dispersed throughout the waste cooking oil at a mass ratio of 1:2. Then, the mixture was sheared at 5000 rpm for 15 min to allow the furfural residue to fully absorb the oil and become more hydrophobic. The extra oil was removed by filtering the material through a 200-mesh filter. The resulting filter cake was referred to as the bio-asphalt, in which the mass ratio of the waste cooking oil to furfural residue was 1.1:1.

#### 2.2.3. Substitution of the Petroleum Asphalt with Bio-Asphalt

Biomass-modified petroleum asphalt was prepared by substituting petroleum asphalt with the previously prepared bio-asphalt at concentrations of 4, 8, and 12 wt.%. After heating in an oven at 140 °C for 2 h, the two materials (petroleum asphalt and bio-asphalt) were slowly mixed by shearing for 30 min at a speed of 5000 rpm. During this process, the temperature was maintained at 140 °C. After completely mixing and shearing, the final asphalt samples were heated at 140 °C for an additional hour before cooling to room temperature.

#### 2.2.4. Characterization of the Bio-Asphalt

The comparison of the crystal structures of the bio-asphalt and furfural residue was enabled by X-ray diffraction (XRD-6100, Shimadzu, Kyoto, Japan). The water-resistance of the bio-asphalt was evaluated using an Contact Angle Meter (OCA20, DataPhysics, Silicon Valley, California, USA). The organic functional groups of the samples were analyzed using a Fourier-transform infrared (FTIR) spectrometer (IN10, ThermoFisher, Waltham, MA, USA) in the attenuated total reflectance (ATR) mode. The microscopic morphologies of the bio-asphalt and furfural residue were observed under a scanning electron microscope (G300, Zeiss, Oberkohen, Germany).

#### 2.2.5. Asphalt Performance

The three major indices (penetration, softening point, and ductility) of the bio-asphalt-modified asphalt samples were evaluated according to the standards ASTM D5, D36, and D113, respectively. Take 50 g of melted sample and pour it into an aluminum tube with a diameter of 25 mm and a length of 140 mm. Close the tube opening, put it into an oven at 163 ± 5 °C and stand vertically for 6, 12, 24, 48 and 72 h, respectively. Then, take out the sample and put it vertically into the refrigerator for 4 h, respectively, within the corresponding times. After the asphalt solidifies, cut the aluminum tube into three equal parts, take the asphalt in the upper and lower sections, and test the softening point according to ASTM D 36. The softening point difference between the upper and lower sections can tell the storage stability of asphalt. The rheological properties when the asphalt samples were at high temperatures were determined according to the standard AASHTO T315. The phase angle (δ) and complex shear modulus (G*) of the asphalt samples were measured over the angle range of 40–90 °C using a dynamic shear rheometer (MCR302, Anton Paar, Graz, Austria) at a heating rate of 2 °C/min. The flexural creep stiffness at low temperatures was evaluated according to the standard AASHTO T313. The low temperature performance of the asphalt was determined using a bending beam rheometer (TE-BBR SD, Cannon, Los Angeles, USA) at −18 °C, −24 °C, and −30 °C. The results were discussed in the parameters of stiffness modulus (S) and creep rate (m). The furfural residue and the waste cooking oil were also tested in parallel for comparison.

#### 2.2.6. Road Performance of the Substituted Asphalt

The aggregate gradation of AC16 is shown in Table 1. According to the test results of mix design, it was concluded that the best asphalt content in the asphalt-stone mixture with different substituted asphalts (0%, 4%, 8%, and 12%) is 4.9%, 5.1%, 5.3%, and 5.6%, respectively. The road performances of the substituted asphalt, such as high-temperature stability, low-temperature crack resistance, and water stability, were evaluated by conducting rutting tests, low-temperature bending tests, immersion Marshall stability tests, and freeze-thaw splitting tests, respectively.

#### 2.2.7. Statistical Analysis

Experiments for penetration, ductility, softening point, hot storage stability, low-temperature bending creep stiffness, high-temperature stability, low temperature crack resistance, and water stability were performed in triplicate. IBM SPSS Statistics 25 software was used for conducting statistical analysis. All statistics were presented as the mean or the mean ± standard deviation). Significant differences among different groups and within the sample group were performed statistically by one-way analysis of variance (ANOVA). Differences at *p* < 0.05 were considered to be significant. The analysis results are shown in the figures.

## 3. Results

### 3.1. Raw Materials Analysis

#### 3.1.1. Furfural Residue Analysis

**Figure 2:**
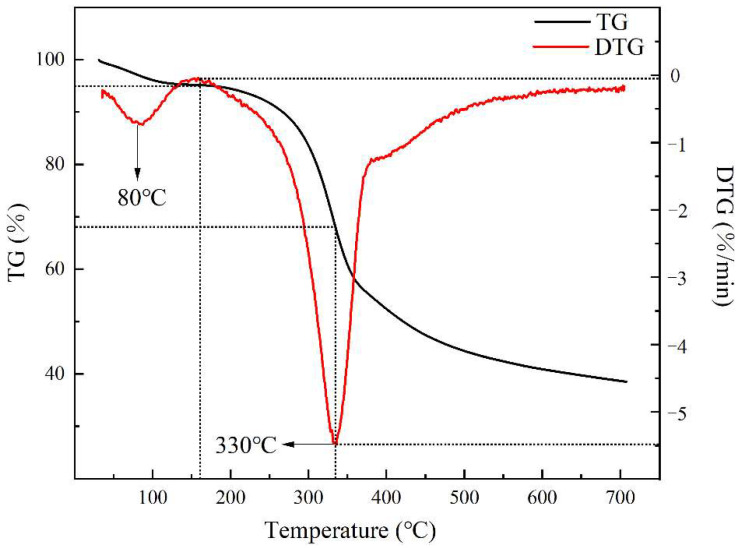
TG and DTG curves of the furfural residue.

Due to lack of hemicellulose, furfural residue is less hydrophilic than other plant fibers. The water absorption rate of the dried furfural residue powder was only 13% after storing at 20 °C and at 90% relative humidity (RH) for 3 days. The thermogravimetric (TG) and differential thermogravimetry (DTG) curves of the furfural residue are shown in Figure 2. In the TG curve, the first weight loss peak of furfural residue was very weak and appeared from 80 °C to 160 °C. Across this range, a weight loss of approximately −0.5%/min occurred, which was mainly attributed to the evaporation of free water in the sample. Following this, rapid cracking of the furfural residue occurred. In the DTG curve of the furfural residue, a maximum weight loss peak appeared at 330 °C, at which a weight loss of approximately –5.5%/min occurred. These results indicated that the thermal stability of the furfural residue was highest below 160 °C.

#### 3.1.2. Waste Cooking Oil Analysis

Raw material waste cooking oil was brown in color and had a pH of 6.8 and a density of 0.943 g·mL^−1^. The results showed that the TPM content of waste cooking oil has reached 30.28%. According to the Chinese standard GB 2716–2018 (2018), the content of TPM in frying oil should not exceed 27%.

#### 3.1.3. 90# Petroleum Asphalt Analysis

The technical specifications and standard requirements of the 90# petroleum asphalt used in this study are shown in Table 2.

### 3.2. Characterization

#### 3.2.1. X-ray Diffraction Analysis of the Bio-Asphalt

**Figure 3:**
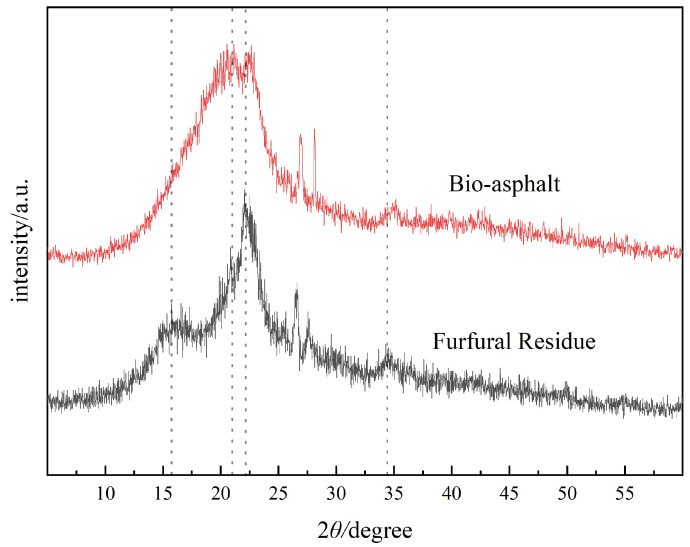
XRD patterns of the furfural residue and the bio-asphalt.

The XRD patterns of the furfural residue and bio-asphalt are shown in Figure 3. The XRD spectrum of the furfural residue featured obvious diffraction peaks at 16.3° and 22.5°, which represented the (101) and (002) diffraction planes of the cellulose crystals, indicating that the cellulose in the furfural residue existed in the cellulose Ⅰ form. After the furfural residue fully absorbed the oil and the mixture was sheared at 5000 rpm for 15 min, the diffraction peak at 16.3° disappeared completely, and the diffraction peak that centered at around 22.5° broadened significantly. These results indicated that the shearing process in the oil caused more of the hydrophobic crystal surface to be exposed, suggesting that the hydrophobicity of furfural residue increased [22].

#### 3.2.2. Water Contact Angle of the Bio-Asphalt

**Figure 4:**
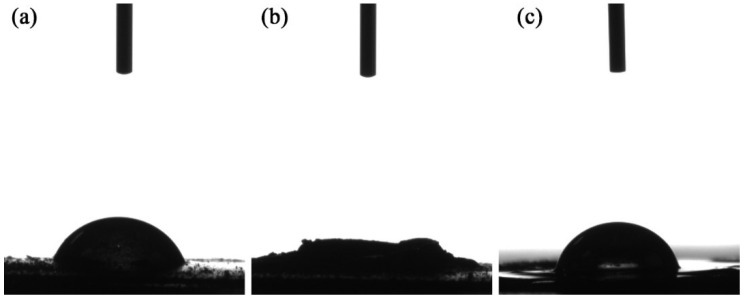
Images of the water contact angle experiments with the furfural residue and bio-asphalt. (**a**) water droplet after immediately falling onto the surface of the furfural residue, (**b**) water droplet after one second of contact with the surface, (**c**) water droplet of the bio-asphalt.

The results of the water contact angle tests are shown in Figure 4a depicts a water droplet after falling onto the surface of the furfural residue, and its water contact angle was 60.2°. After one second, the water droplet was totally absorbed by the furfural residue (Figure 4b); however, after the furfural residue fully absorbed the oil and the mixture was sheared, the water contact angle was 68.6° (Figure 4c). This angle could be maintained in this state; therefore, these results demonstrated that the hydrophobicity was significantly improved over the furfural residue.

#### 3.2.3. FTIR Analysis

**Figure 5:**
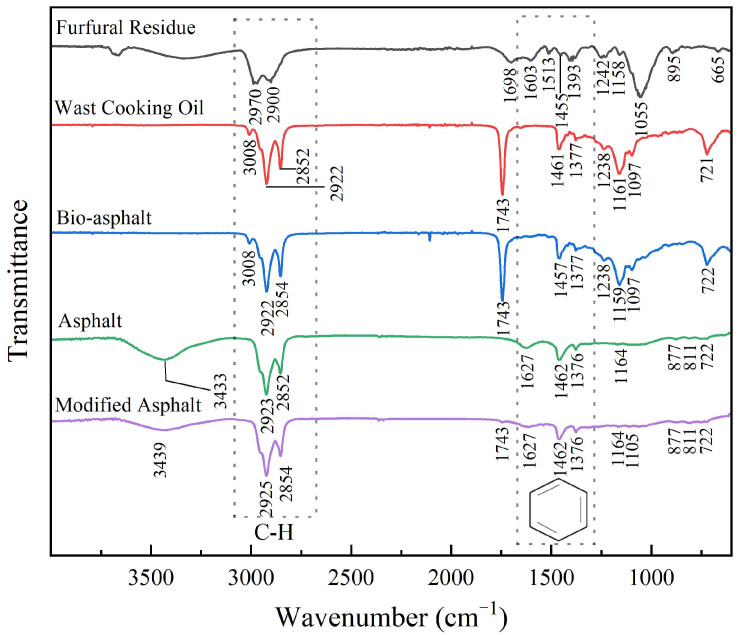
Infrared spectra of the samples.

The organic functional groups on the surface of the various materials utilized in this study were surveyed by FTIR spectroscopy using a spectrometer (IN10, Thermo Fisher, Waltham, MA, USA) equipped with an ATR accessory (Figure 5). Furfural residue contains significant amounts of cellulose and lignin, with small amounts of hemicellulose. This was corroborated by the presence of methyl and methylene C–H vibration absorption peaks at 2970 cm^−1^ and 2900 cm^−1^ and characteristic ester and carbonyl absorption peaks at 1698 cm^−1^. The absorption peaks at 1603 cm^−1^, 1513 cm^−1^, and 1455 cm^−1^ represented the characteristic peaks of lignin, indicating that the lignin structure maintained its integrity [23]. The strong absorption peak at 1055 cm^–1^ corresponded to the C–O–C stretching vibration of the β-1,4 glycosidic bond of cellulose [24].

The main components of the waste cooking oil are carboxylic acids, fatty acids, alkanes, and triglycerides. In the FTIR spectrum of the waste cooking oil (Figure 5, red curve), strong absorption peaks at 2922 cm^−1^ and 2852 cm^−1^ represented the vibrations of the C–H bonds of the alkanes and cycloalkanes. The absorption peaks at 1743 cm^−1^ and 1161 cm^−1^ corresponded to the C=O bonds of the carboxylic acid groups of the fatty acids, whereas the absorption bands at 1461 cm^−1^ and 1377 cm^−1^ were attributed to the vibrations of the asymmetric C–CH_3_ bonds [25], indicating that there were saturated hydrocarbons in the waste cooking oil. Furthermore, the absorption band at 721 cm^–1^ was attributed to the vibrations of the C–H bonds in the (CH_2_) plane [26]. As the ATR accessory was used to collect the infrared spectra, only the spectral information of the surface of the materials was obtained. The FTIR spectrum of the bio-asphalt (Figure 5, blue curve) was consistent with the FTIR spectrum of the waste cooking oil, and no new absorption peaks were observed, indicating that the mixing of the furfural residue and waste cooking oil comprised mainly physical mixing.

The chemical structure of asphalt is complex and contains asphaltene, aromatics, resins, and a saturated oil fraction. The FTIR spectrum of the petroleum asphalt (Figure 5, green curve) featured a broad and weak absorption peak centered at 3433 cm^−1^, which might have been caused by intermolecular hydrogen bonds between the hydroxyl groups of asphalt, forming a polymer-like network. Analogous to the waste cooking oil, the FTIR spectrum of the asphalt featured strong absorption peaks at 2923 cm^−1^ and 2852 cm^−1^, which were attributed to the stretching vibrations of the C–H bonds of alkanes and naphthalenes [27], indicating that there were saturated hydrocarbons in the asphalt. The absorption peak at 1627 cm^−1^ was associated with the aromatic C=C bonds and C=O bonds [28], indicating that there were aromatic compounds in the asphalt. The asymmetric vibrations of the C–CH_3_ bonds at 1462 cm^−1^ and 1376 cm^−1^ also corroborated the presence of saturated hydrocarbons in the asphalt. The absorption bands at 877 cm^−1^, 811 cm^−1^, and 722 cm^−1^ were attributed to aromatic out-of-plane vibrations, which further corroborated the presence of aromatic compounds in the asphalt [12]. Compared with the FTIR spectrum of the bio-asphalt, no new absorption peaks were observed in the FTIR spectrum of biomass-modified asphalt (Figure 5, purple curve), indicating that the biomass-modified asphalt was prepared mainly by physically mixing the bio-asphalt and petroleum asphalt ingredients, and there were no obvious chemical reactions between the components.

#### 3.2.4. Morphology Analysis

**Figure 6:**
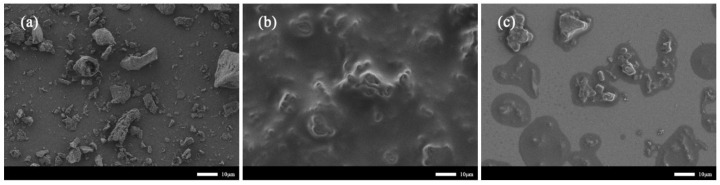
SEM images of the (**a**) furfural residue, (**b**) bio-asphalt, and (**c**) partially substituted asphalt.

Figure 6 shows the microscopic morphologies of the furfural residue (a), bio-asphalt (b), and the biomass-substituted asphalt samples (c). The furfural residue particles were irregular in shape and size, and their surfaces were very rough, which enabled them to form strong interfacial interactions with the components in the waste cooking oil to form a stable composite system (Figure 6a). The images in Figure 6b,c show that the furfural residue was uniformly dispersed throughout waste cooking oil and asphalt system, respectively, indicating that the three matrices were highly compatible with each other.

### 3.3. Mechanical Performance of the Asphalt Mixtures

#### 3.3.1. Penetration, Softening Point, and Ductility

The penetration, softening point, and ductility of asphalt are important indices for characterizing the basic performance of asphalt. Penetration is closely related to viscosity, and can reflect the consistency, softness, and hardness of the material at 25 °C. The softening point is representative of the fluidity of the asphalt reaching the critical point of a particular phase while being heated, and it can reflect the stability of asphalt at high temperatures. Ductility represents the ability of a material, in this case asphalt, to withstand tensile deformation at low temperatures. In this work, the penetration, softening point, and ductility of the various asphalt samples were measured.

**Figure 7:**
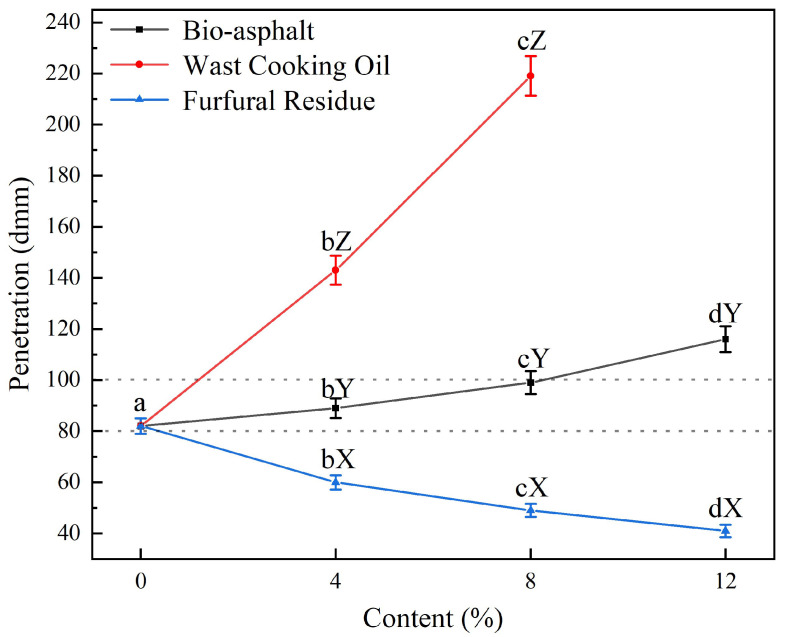
Effects of modifying petroleum asphalt with varying contents (0–12 wt.%) of furfural residue (blue data), waste cooking oil (red data), and bio-asphalt (black data) on the penetration of the resulting biomass-modified petroleum asphalt samples. Different lowercase letters indicate significant differences (*p* < 0.05) between different contents of the same modifier; different uppercase letters indicate significant differences (*p* < 0.05) between different modifiers of the same content.

The results of the penetration tests are shown in Figure 7. As the concentration of bio-asphalt in the biomass-modified petroleum asphalt increased from 0 wt.% to 12 wt.%, the penetration of the resulting biomass-modified asphalt mortar increased from 82 dmm to 116 dmm. When the content was 8 wt.%, the penetration of the asphalt was still below 100 dmm, which was in accordance with the technical requirements of 90# petroleum asphalt in the Technical Code for Highway Asphalt Pavement Construction that requires the penetration of asphalt to be in the range of 80–100 dmm. The addition of waste cooking oil significantly increased the penetration of the asphalt mortar to be much higher than when bio-asphalt was added. On the contrary, the furfural residue reduced the penetration of the asphalt mortar. When the asphalt was modified with only 4 wt.% furfural residue, the penetration of the resulting modified asphalt was too low to meet the standard requirements. These results indicated that the addition of either waste cooking oil or the bio-asphalt reduced the consistency and deformation resistance of petroleum asphalt, whereas the addition of furfural residue improved the consistency of the asphalt. Furfural residue has the ability to absorb some of the oil in asphalt and undergo cross-linking with asphaltene in the asphalt to form an interconnected network structure, thereby increasing its hardness. In the near future, bio-asphalt featuring higher ratios of furfural residue to waste cooking oil should be prepared and used to partially substitute petroleum asphalt to increase the mechanical strength of the asphalt.

**Figure 8:**
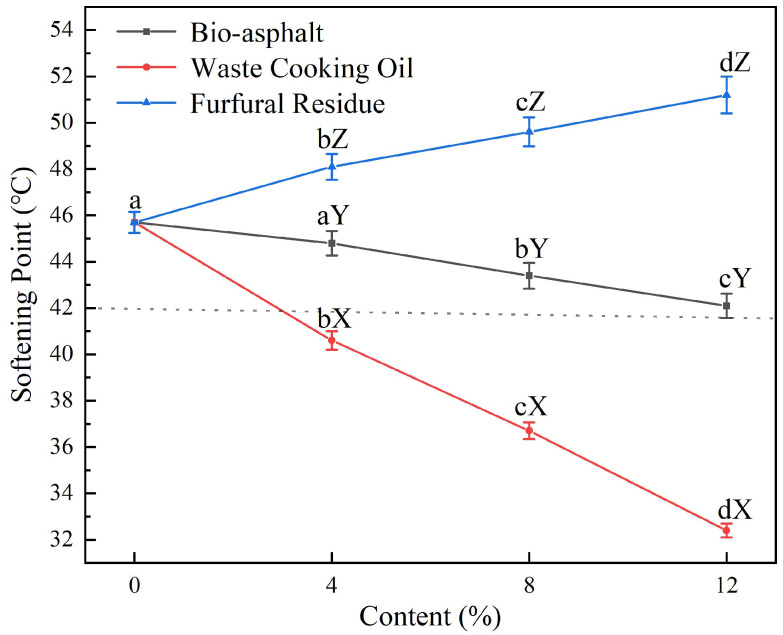
Effects of modifying petroleum asphalt with varying contents (0–12 wt.%) of furfural residue (blue data), waste cooking oil (red data), and bio-asphalt (black data) on the softening point of the resulting biomass-modified petroleum asphalt samples. Different lowercase letters indicate significant differences (*p* < 0.05) between different contents of the same modifier; different uppercase letters indicate significant differences (*p* < 0.05) between different modifiers of the same content.

Figure 8 shows the effects of modifying petroleum asphalt with varying contents (0–12 wt.%) of furfural residue (blue curve), waste cooking oil (red curve), and bio-asphalt (black curve) on the softening point of the resulting biomass-modified petroleum asphalt samples. The asphalt softening point decreased with increasing bio-asphalt content (black curve). Although the softening point of the asphalt mortar decreased to 42.1 °C when the bio-asphalt content increased to 12 wt.%, the bio-asphalt-modified asphalt mixtures still met the technical requirements of 90# road petroleum asphalt in the Technical Code for Highway Asphalt Pavement Construction. The addition of waste cooking oil to the petroleum asphalt made the softening point of the resulting asphalt mortar drop sharply (red curve). When the waste cooking oil content was 4 wt.%, the softening point of the asphalt mortar was 40.0 °C, which was too low to meet the standard requirements. On the contrary, the addition of furfural residue to the petroleum asphalt significantly increased the asphalt softening point (blue curve) from 45.7 °C to 51.2 °C as the content increased from 0 to 12 wt.%. These results showed that the waste cooking oil and the bio-asphalt adversely affected the high-temperature stability of the asphalt, whereas the furfural residue significantly improved the high-temperature stability of the asphalt.

**Figure 9:**
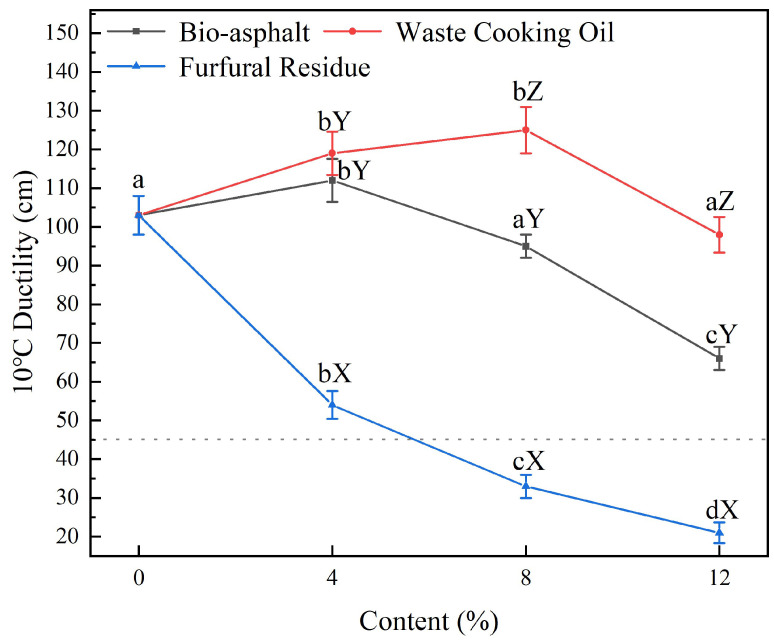
Effects of modifying petroleum asphalt with varying contents (0–12 wt.%) of furfural residue (blue data), waste cooking oil (red data), and bio-asphalt (black data) on the ductility of the resulting biomass-modified petroleum asphalt samples. Different lowercase letters indicate significant differences (*p* < 0.05) between different contents of the same modifier; different uppercase letters indicate significant differences (*p* < 0.05) between different modifiers of the same content.

Figure 9 shows the effects of modifying petroleum asphalt with varying contents (0–12 wt.%) of furfural residue (blue curve), waste cooking oil (red curve), and bio-asphalt (black curve) on the ductility of the resulting biomass-modified petroleum asphalt samples at 10 °C. When the bio-asphalt content was 4 wt.%, the ductility of the asphalt mortar increased from 103 cm to 112 cm, which corresponded to an 8.7% increase; however, although the ductility decreased dramatically to 67 cm as the concentration of the bio-asphalt increased to 12%, a ductility of 67 cm still met the standard requirements of 90# asphalt in the Technical Code for Highway Asphalt Pavement Construction. The addition of waste cooking oil to the petroleum asphalt also improved the ductility of the asphalt. When the waste cooking oil content was 8 wt.%, the ductility was 125 cm, which corresponded to a 21.4% increase over petroleum asphalt. On the contrary, the addition of furfural residue to the petroleum asphalt significantly reduced the asphalt ductility (blue curve) from 103 cm to 21 cm as the content increased from 0 to 12 wt.%. These results indicated that the addition of proper amounts of waste cooking oil (no more than 8%) or bio-asphalt (no more than 4%) to petroleum asphalt could improve the ductility of the asphalt at low temperatures because the waste cooking oil and bio-asphalt increased the fluidity of the asphalt matrix at, or below, room temperature; therefore, the addition of bio-asphalt could improve the flexibility of asphalt at low temperatures, but it also reduces the viscosity of the matrix asphalt and the excessive addition of certain modifiers will adversely affect the tensile properties of the asphalt. Furfural residue reduced the low-temperature performance of petroleum asphalt, likely because furfural residue could absorb components such as oil and resin in the asphalt, thereby reducing its plasticity and fluidity, which lowers the low-temperature ductility of the asphalt. Overall, the bio-asphalt-modified petroleum asphalt had the best overall performance; therefore, in the follow-up test, only the performances of the bio-asphalt-modified asphalt was analyzed and tested in concentrations of 4%, 8%, and 12%.

#### 3.3.2. Thermal Storage Stability

**Figure 10:**
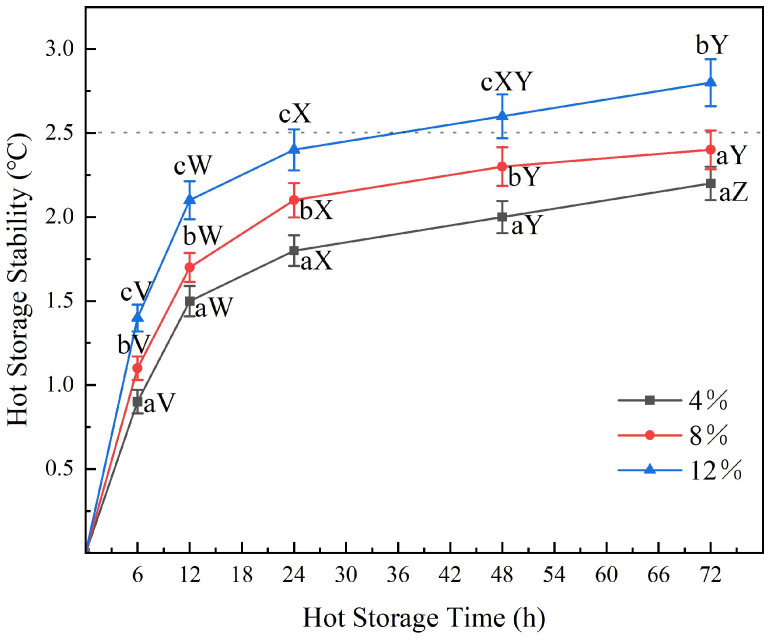
Effects of modifying petroleum asphalt with varying contents (4, 8, and 12 wt.%) of bio-asphalt on the thermal storage stability of the biomass-modified asphalt over a period of 72 h. Different lowercase letters indicate significant differences (*p* < 0.05) between different bio-asphalt contents of the same storage time; different uppercase letters indicate significant differences (*p* < 0.05) of the same bio-asphalt content under different storage times.

There were many similarities in the chemical composition and molecular structure of the bio-asphalt-modified asphalt and the unmodified petroleum asphalt, so they could be mixed effectively without undergoing many chemical changes. Before being applied to the construction, the asphalt needed to be stored at high temperatures for a long time, to ensure that the modifier did not separate from the petroleum asphalt matrix during long-term thermal storage, which would ultimately affect the performance of the asphalt. In this work, the softening point difference method was used to analyze the high-temperature stability of the bio-asphalt-modified asphalt samples (containing 4, 8, and 12 wt.% bio-asphalt), and the results are shown in Figure 10. The softening point difference between the upper and lower sections of the substituted asphalt increased as the duration of the thermal storage increased from 0 h to 72 h, but this rate of increase became less significant as time progressed, which indicated that the internal molecular structure of the bio-asphalt-modified asphalt became more stable after undergoing slight separation of the components at high temperature. Higher concentrations of the bio-asphalt manifested larger asphalt softening point differences; however, the high-temperature stability of the modified asphalt featuring a bio-asphalt content of 8 wt.% over the 72 h period was still enough to meet the requirements of the standard. These results demonstrated that the bio-asphalt had excellent compatibility with the matrix asphalt.

#### 3.3.3. High Temperature Rheological Properties

**Figure 11:**
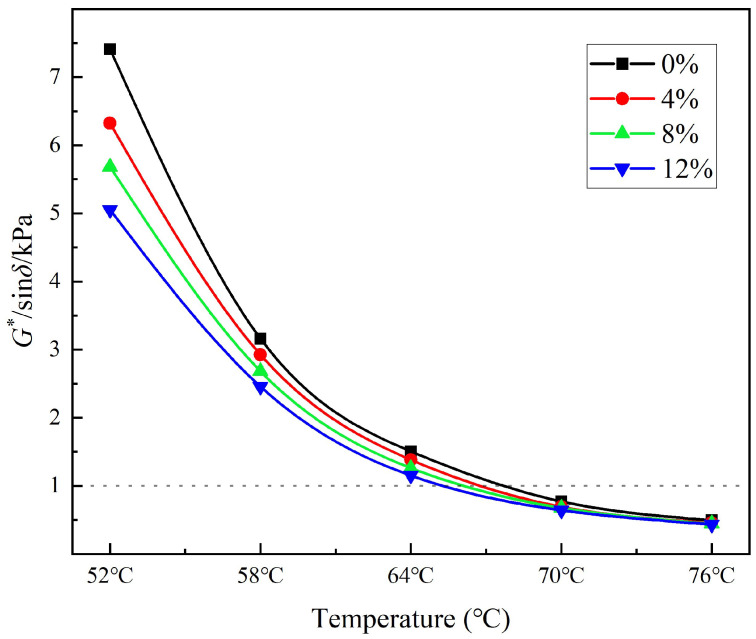
Effect of the bio-asphalt content on the rutting factor of the modified asphalts.

The high-temperature performances of the modified asphalts were evaluated using a dynamic shear rheometer (DSR), and their high-temperature stabilities were evaluated based on the rutting factor G*/sin δ. The higher the value, the stronger the permanent deformation resistance of the asphalt at a given temperature; therefore, the better the rutting resistance. Figure 11 shows the effect of different bio-asphalt contents on the rutting factor of the modified asphalts. The rutting factor decreased with increasing bio-asphalt content at each temperature evaluated, indicating that the high temperature deformation resistance became worse as the bio-asphalt content increased. When the bio-asphalt content was 12 wt.%, the rutting factor at 64 °C was greater than 1 kPa; however, when the temperature increased to 7 °C and 76 °C, the rutting factors of all three bio-asphalt-modified petroleum asphalt samples became more consistent (all less than 1 kPa) than at lower temperatures.

#### 3.3.4. Low-Temperature Bending Creep Stiffness

**Figure 12:**
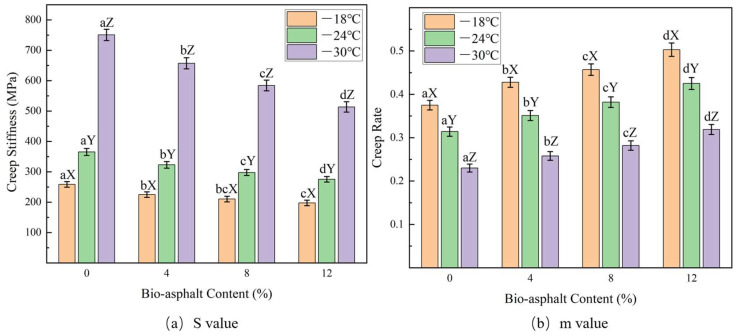
Effects of the bio-asphalt content on (**a**) bending creep stiffness (S value) and (**b**) creep rate (m value) of the modified petroleum asphalts at low temperatures. Different lowercase letters indicate significant differences (*p* < 0.05) between different bio-asphalt contents under the same temperature; different uppercase letters indicate significant differences (*p* < 0.05) of the same bio-asphalt content under different temperatures.

The low-temperature crack resistance of the modified asphalt samples was evaluated using a bending beam rheometer (BBR), and their low temperature performances were evaluated based on the creep stiffness (S) and creep rate (m). Smaller S values and higher m values were indicative of a better low-temperature crack resistance of the asphalt. The results of the BBR tests of the three partially bio-asphalt-substituted petroleum asphalt samples are shown in Figure 12. When the bio-asphalt contents were the same, the S value of the modified asphalt increased as the temperature decreased (Figure 12a), indicating that it was easier to generate thermal stress inside the asphalt at lower temperatures, which led to cracking of the material. At each of the temperatures evaluated, the S value of asphalt decreased as the bio-asphalt content increased, indicating that the addition of modifiers reduced the internal temperature stress of the asphalt and reduced the risk of the material cracking under extremely cold conditions. When the bio-asphalt content was held constant, the creep rate of the modified asphalt decreased with the decreasing temperature (Figure 12b), indicating that it was more difficult for the modified asphalts to relax at lower temperatures. When the temperature was held constant, the m value of modified asphalt increased with increasing bio-asphalt content, indicating that the addition of the bio-asphalt enhanced the stress dissipation capacity of the petroleum asphalt at low temperatures.

According to the requirements of the American Superpave asphalt specification, the S value of an asphalt material at the design temperature should not be greater than 300 MPa, and the m value should not be less than 0.3. From the results shown in Figure 12, the S values and m values of all four bio-asphalt-modified petroleum asphalts at −18 °C met the requirements of the specification. When the temperature dropped to −24 °C, although the S values of the unmodified petroleum asphalt (0%) and the 4% bio-asphalt sample were slightly lower than the standard requirements, all other values of the unmodified and modified asphalts met the specification requirements. When the temperature was lowered further to −30 °C, all S values and m values did not meet the requirements of the specification, except for the m value of the asphalt modified with 12 wt.% bio-asphalt; therefore, these results indicated that bio-asphalt could improve the low-temperature crack resistance of petroleum asphalt, especially at higher bio-asphalt contents (within a certain range).

### 3.4. Road Performance

#### 3.4.1. High-Temperature Stability

**Figure 13:**
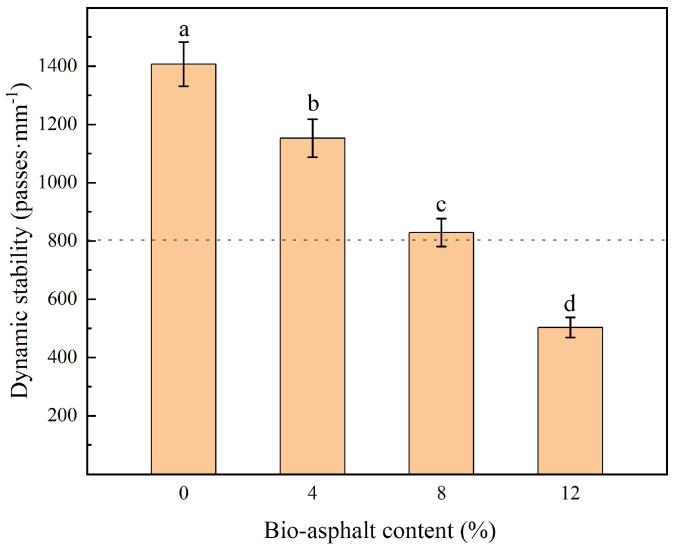
Effect of the bio-asphalt content on the dynamic stability of the asphalt mixtures. Different lowercase letters indicate significant differences (*p* < 0.05) between different bio-asphalt contents.

The high-temperature stability of an asphalt mixture refers to the ability of the asphalt mixture to resist some permanent deformation, such as rutting, moving, and swelling, when exposed to hot weather. In this work, rutting tests were used to determine the high-temperature stabilities of the modified asphalt mixtures, and the results are shown in Figure 13. The dynamic stability of the substituted asphalt mixtures decreased gradually from 1407 to 503 times/mm as the bio-asphalt content increased from 0 wt.% to 12 wt.%. This change amounted to a lower dynamic stability compared with petroleum asphalt, at 41% and 64.3%, respectively. These results indicated that, although the substitution of a portion of the petroleum asphalt with bio-asphalt resulted in lower rutting resistances, the modified petroleum asphalt samples with bio-asphalt contents of 8 wt.% and lower had rutting resistances that were in accordance with the standard requirements.

#### 3.4.2. Low Temperature Crack Resistance

**Figure 14:**
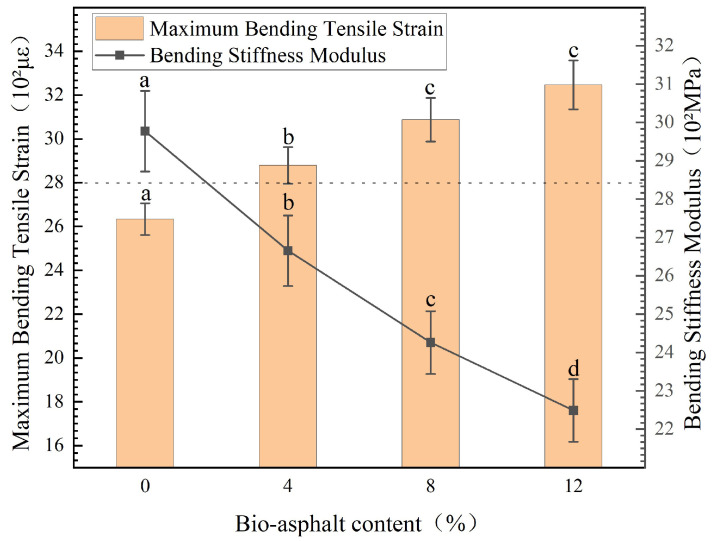
Effect of the bio-asphalt content on the low-temperature crack resistance of the biomass-modified asphalt mixtures. Different lowercase letters indicate significant differences (*p* < 0.05) between different bio-asphalt contents.

At low temperatures, the tensile strength and deformability of asphalt will decrease significantly, making the asphalt more prone to cracking because of the decreased cracked resistance. In this work, the low-temperature crack resistance of the bio-asphalt-modified asphalt mixtures was evaluated through low-temperature bending tests, the results of which are shown in Figure 14. The maximum bending strain of the asphalt mixtures increased from 2633.32 με (0 wt.% bio-asphalt) to 2879.25 με, 3087.44 με, and 3246.13 με, for the modified asphalt mixtures with bio-asphalt contents of 4%, 8%, and 12%, respectively, which corresponded to increases of 9.3%, 17.2%, and 23.3%, respectively. The low-temperature bending stiffness modulus of the modified asphalt mixtures decreased gradually as the bio-asphalt content increased from 0 wt.% to 12 wt.%. When the bio-asphalt content was 12 wt.%, the bending stiffness modulus decreased by 24.5% compared with the unmodified petroleum asphalt. These results demonstrated that the low-temperature crack resistance of the modified asphalt mixture increased with increasing bio-asphalt content.

#### 3.4.3. Water Stability

**Figure 15:**
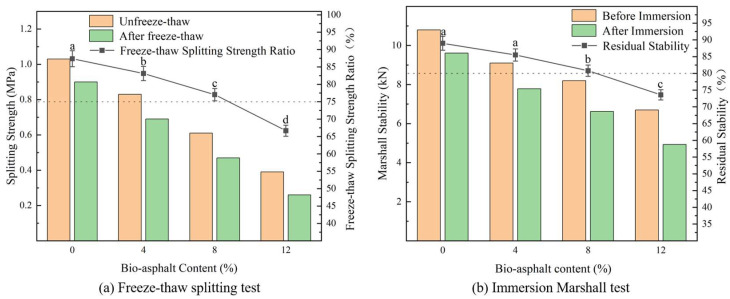
Effect of bio-asphalt content on (**a**) freeze-thaw splitting strength ratio and (**b**) residual stability of the biomass-modified asphalt mixtures. Different lowercase letters indicate significant differences (*p* < 0.05) between different bio-asphalt contents.

After slow but continuous erosion by rainwater and damage by repeated freeze-thaw cycles and vehicle rolling, the adhesion of the interface between asphalt and the aggregates becomes weakened, allowing moisture to penetrate the asphalt and further erode the bonding surfaces within the asphalt, resulting in a separation of the mixture, loosening, and cracking; therefore, we assessed the water stability of the biomass-modified asphalt mixtures by conducting immersion Marshall stability tests and freeze-thaw splitting tests, the results of which are shown in Figure 15. The freeze-thaw splitting strength ratio in the figure refers to the splitting strength of frozen thawed specimen divided by the splitting strength of the unfrozen thawed specimen. Residual stability in the figure refers to the Marshall stability after immersion treatment divided by the untreated Marshall stability. Overall, the freeze-thaw splitting strength ratio and residual stability of the various asphalt mixtures decreased with increasing bio-asphalt content. The freeze-thaw splitting strength ratio of the asphalt mixtures decreased from 87.4% (unmodified petroleum asphalt) to 83.1%, 77%, and 66.7% for the biomass-modified asphalt mixtures with bio-asphalt contents of 4%, 8%, and 12%, respectively, which corresponded to decreases of 4.9%, 11.9%, and 23.7%, respectively. Furthermore, the residual stability decreased from 89% (petroleum asphalt) to 85.5%, 80.8%, and 73.6%, respectively, which corresponded to decreases of 3.9%, 9.2%, and 17.3%, respectively. These results showed that substitution of a portion of the petroleum asphalt with bio-asphalt could reduce the water stability of the asphalt, but these reductions were not obvious at lower bio-asphalt contents. Nonetheless, the biomass-modified asphalt mixtures still met the standard requirements when the bio-asphalt content was no more than 8 wt.%.

## 4. Conclusions

In this study, bio-asphalt was prepared by physically mixing furfural residue and waste cooking oil (soybean oil) as raw materials. Petroleum asphalt was partially substituted with varying concentrations of the low hydrophilic bio-asphalt from 0 wt.% to 12 wt.%. Based on the performance measurement of biomass-modified asphalt, the following conclusions can be drawn:

(1) The addition of bio-asphalt increased the penetration of asphalt and reduced its softening point, and its ductility value increased at first and then decreased with the increase of substitution amount.
(2) The bio-asphalt exhibited excellent compatibility with 90# asphalt, when the bio-asphalt substitution amount was 8 wt.%, its 72 h hot storage stability met the standard requirements of 90# petroleum asphalt.
(3) Substitution of petroleum asphalt with bio-asphalt had an adverse effect on the high-temperature rutting resistance performance and water stability of the resulting asphalt.
(4) The low-temperature stress relaxation performance and low-temperature crack resistance increased as the bio-asphalt content increased.
(5) When the bio-asphalt content was no more than 8 wt.%, the road performance indices of the biomass-modified asphalt met the standard requirements of 90# petroleum asphalt. The asphalt modified with the bio-asphalt was more suitable for use in low-temperature conditions than for use in high-temperature conditions.

## Figures and Tables

**Table 1:** Mineral Aggregate Gradation of AC-16 Asphalt Mixture.

Sieve Pore Size/mm	Passing Percentage		
	Upper Limit of Gradation	Aggregate Gradation	Lower Limit of Gradation
19	100	100	100
16	100	98.5	90
13.2	92	86	76
9.5	80	68	60
4.75	62	47	34
2.36	48	31	20
1.18	36	21	13
0.6	26	14	9
0.3	18	9.5	7
0.15	14	7.2	5
0.075	8	5	4

**Table 2:** Technical specifications and standard requirements of 90# petroleum asphalt.

Test Project	Measured Value	Standard Requirements
Penetration degree (25 °C, 5 s,100 g)	83 dmm	80~100 dmm
Softening point	45.7 °C	≥42 °C
Ductility (10 °C)	103 cm	≥15 cm
Ductility (15 °C)	>150 cm	≥50 cm

## Data Availability

Not applicable.
